# The Dissector, or Practical and Surgical Anatomy

**Published:** 1844-04

**Authors:** 


### BIBLIOGRAPHICAL NOTICES.

*The Dissector, or Practical and Surgical Anatomy.* By ERASMUS WILSON, author of a "System of Human Anatomy," &c., with one hundred and six Illustrations, modified and re-arranged by PAUL B. GODDARD, M. D., Demonstrator of Anatomy, in the University of Pennsylvania. Philadelphia: Lee & Blanchard, 1844, p. 444, 8vo.

We take pleasure in recommending the above work to the attention of Physicians and Medical Students. For accuracy of description, comprehensiveness, and conciseness of style, it takes precedence over all the works of practical anatomy in common use, and ranks with the very first with which we are accquainted. The introduction of surgical, in connection with practical anatomy, into a "dissector," is an arrangement, the utility of which might be questioned by some, but this part of the present work occupies but a small space, and is well calculated to give interest to the descriptions as well as to convey useful information. But the illustrations are the part of the book which will render it a favorite with students, and contribute most essentially to its usefulness. They may be compared to the figures on the black board, the utility of which in anatomical descriptions, especially of the vascular and nervous systems, is beginning to be appreciated, by teachers of that science. The execution of the mechanical part is excellent. We should be glad to see introduced into it and into every similar work, formulæ for injections of the vessels, a few directions for making anatomical preparations, and the mode of preserving subjects for dissection, by Gannal's method. This would render it more useful, especially to those engaged in dissections at a distance from medical schools. D. B.

